# Common variable immunodeficiency and natural killer cell lymphopenia caused by Ets-binding site mutation in the IL-2 receptor γ (*IL2RG*) gene promoter

**DOI:** 10.1016/j.jaci.2015.08.049

**Published:** 2016-03

**Authors:** Anita Chandra, Fang Zhang, Kimberly C. Gilmour, David Webster, Vincent Plagnol, Dinakantha S. Kumararatne, Siobhan O. Burns, Sergey Nejentsev, Adrian J. Thrasher

**Affiliations:** a Department of Clinical Biochemistry and Immunology, Cambridge University Hospitals NHS Foundation Trust, Cambridge, United Kingdom; b Lymphocyte Signalling & Development, Babraham Institute, Cambridge, United Kingdom; c Molecular and Cellular Immunology, Institute of Child Health, University College London, London, United Kingdom; d Department of Immunology, Great Ormond Street Hospital NHS Foundation Trust, London, United Kingdom; e University College London Institute of Immunity and Transplantation, London, United Kingdom; f Department of Medicine, University of Cambridge, Cambridge, United Kingdom; g Great Ormond Street Hospital NHS Foundation Trust, London, United Kingdom; h University College London Genetics Institute, University College London, London, United Kingdom; i Department of Immunology, Royal Free London NHS Foundation Trust, London, United Kingdom

To the Editor:

Patients with severe combined immunodeficiency (SCID) of a classical phenotype present within the first year of life with life-threatening infections and failure to thrive.^1^ The X-linked T^−^B^+^ natural killer (NK)^−^ form of SCID is the most frequent type (44% to 46%) and is a consequence of mutations in the IL-2 receptor γ *(IL2RG)* gene (OMIM 308380), which encodes the common cytokine receptor γ chain (γc).^2^ The γc acts as a signal-transducing subunit of cytokine receptors that are essential in the ontogeny and function of T, B, and NK cells, namely IL-2, IL-4, IL-7, IL-9, IL-15, and IL-21. The intracellular part of γc interacts with Janus kinase 3 and mediates phosphorylation and activation of signal transducer and activator of transcription (STAT) proteins, which regulate induction of gene transcription.

A number of patients with a milder form of combined immunodeficiency, often termed “leaky” or “hypomorphic” SCID, have been described. Here we describe 2 male relatives with a novel hypomorphic mutation in the *IL2RG* promoter who presented with a phenotype more akin to common variable immunodeficiency (CVID). CVID is the most common clinically and genetically heterogeneous primary immunodeficiency, which is characterized by low IgG, IgA, and/or IgM levels, with a failure to produce specific antibodies.^3^ Mutations in genes encoding transmembrane activator and CAML interactor (TACI), inducible costimulator (ICOS), CD19, CD20, CD21, CD81, LRBA, CXCR4, NF-κB2, B cell–activating factor of the TNF family (BAFF) receptor, TNF-related weak inducer of apoptosis (TWEAK), phosphoinositide 3-kinase catalytic subunit δ polypeptide (PI3KCD), and PI3KR1 were shown to cause CVID-like phenotypes.^3^

The grandson presented at age 4 years with a history of recurrent bacterial otitis media and chronic suppurative rhinitis, rotavirus-induced gastroenteritis (age 18 months), echoviral gastroenteritis (age 2 years), and varicella zoster (age 4 years). He had IgG deficiency (1.8 g/L) with normal IgA and IgM levels (1.0 and 0.5 g/L, respectively) and did not mount an adequate response to the 23-valent pneumococcal polysaccharide vaccine (Pneumovax; Merck & Co, Whitehouse Station, NJ), although he responded appropriately to immunization with protein antigens (see Table E1 in this article's Online Repository at www.jacionline.org). He had normal numbers of T and B cells but completely absent NK cells. T-cell proliferation after stimulation with PHA, anti-CD3, and *Candida* species was suboptimal but not completely abrogated. He was started on immunoglobulin replacement therapy and is well, with his infections limited to recalcitrant cutaneous warts.

At the time of his diagnosis, it was noted that his maternal grandfather was under treatment for CVID. The grandfather presented to an immunology team at the age of 34 years with a 20-year history of recurrent otosinopulmonary tract infections with *Streptococcus pneumoniae* and *Haemophilus influenzae*, bronchiectasis, and type 1 diabetes mellitus and celiac disease. On initial presentation, he had an IgG_2_ and IgG_4_ subclass deficiency, absent antibody response to polysaccharide vaccine, CD4 and NK lymphopenia, and reduced proliferative responses to PHA. Immunoglobulin substitution was implemented, along with antibiotic prophylaxis, and he was managed successfully on this regimen for 25 years until he died at age 62 years after a cardiac event.

Flow cytometric analysis of lymphocytes revealed a significantly diminished γc expression in both the grandson and grandfather (Fig 1, *A*; see the Methods section in this article's Online Repository at www.jacionline.org). Likewise, *IL2RG* mRNA expression in sorted T and B cells from the grandson showed a 4.2-fold reduction in T cells and a 33-fold reduction in B cells compared with healthy control subjects (Fig 1, *B*). The T cells were then stimulated with IL-2, IL-7, and IL-15, and phosphorylated STAT5 levels were determined by means of flow cytometric analysis (Fig 1, *C*). This was diminished in the patient with a fold reduction compared with healthy control samples of 3.5-, 7.5-, and 3.8-fold for IL-2, IL-7, and IL-15, respectively.

X-inactivation studies performed on samples from the mother of the grandson demonstrated random X-inactivation in whole blood but apparent nonrandom X-inactivation in T cells (see Table E2 in this article's Online Repository at www.jacionline.org). Whole-exome sequencing of the grandson revealed a point mutation, C to T at position g.chrX:71,111,618 (GRCh38), which was located −13 nucleotides upstream of the transcription start site in the *IL2RG* gene (ENST00000374202; Fig 1, *D*). This is situated at an identified binding site for the transcription factor ETS, which is required for basal promoter activity in cell lines.^4^

For functional validation, we generated the same mutation in an *IL2RG* minigene (Mut.gcPRO and WT.gcPRO), and using a lentiviral vector, introduced this into γc-deficient ED7R cells. We found that γc expression from Mut.gcPRO was dramatically abrogated when compared with the wild-type sequence in a dose-dependent manner. When transduced at similar efficiency (similar vector copy numbers), there is an 8-fold difference in γc expression between the WT-gcPRO and Mut-gcPRO transduced cells (Fig 1, *E*). To confirm that the mutation abrogated binding of ETS, using the electrophoretic mobility shift assay, we showed that mutant oligonucleotides were unable to form a normal protein/DNA complex (Fig 2).

More than 100 mutations in *IL2RG* have been described extending across all of its 8 exons, intron/exon boundaries, and 3′ regulatory regions.^4^ Although most of the known mutations result in a classical immunophenotype of T^−^B^+^NK^−^ SCID, variants leading to a T^−^B^+^NK^+^ SCID and T^low^B^+^NK^+^ have been described.^5^, ^6^ Attenuated SCID phenotypes have also been observed as a result of splice-site mutations resulting in diminished expression of truncated γc protein or as a result of somatic reversion.^5^, ^6^, ^7^

Here we identified a novel point mutation at nucleotide −13 upstream of the transcription start site in a putative ETS-binding site.^3^ ETS transcription factors comprise a large evolutionarily conserved family characterized by sequence homology within their DNA-binding domain that bind to sequences containing a consensus GGAA/T motif.^8^ The ETS transcription factors have been linked with diverse biological processes, including hematopoiesis, T-cell survival, and NK cell production.^9^ Previous studies have shown that an ETS-binding site in a 1053-bp fragment 5′ to the *IL2RG* transcription initiation site is essential for tissue-specific basal promoter activity of *IL2RG*.^3^

Our data indicate that a point mutation within the ETS-binding site of the proximal *IL2RG* promoter has a significant detrimental effect on its activity in human subjects. The residual expression of γc appears to differentially affect signaling through the cytokine receptors leading to normal T-cell development, with minimal reduction in T-cell function and absent NK cell development. In this family this resulted in an initial presentation akin to CVID, manifesting with recurrent bacterial and viral infections. This scenario should be considered in male patients with antibody deficiency, particularly if accompanied by NK lymphopenia. These patients should also be monitored closely for more serious manifestations because this defect is amenable to correction by means of hematopoietic stem cell transplantation or gene therapy. Furthermore, our finding highlights the potential role of mutations in gene regulatory regions as a cause of significant primary immunodeficiencies.

## Figures and Tables

**Table E1:** Summary of immunologic investigations in the patients

Investigation	Grandson 44 y (normal range)	Grandson 4 y (normal range)
CD3^+^ cells (10^9^/L)	0.83 (0.7-2.1)	4.34 (0.9-4.5)
CD19^+^ cells (10^9^/L)	0.01 (0.1-0.5)	1.10 (0.2-2.1)
CD16^+^CD56^+^ cells (10^9^/L)	0.00 (0.09-0.6)	0.00 (0.1-1.0)
CD3^+^CD4^+^ cells (10^9^/L)	0.25 (0.3-1.4)	2.86 (0.5-2.4)
CD3^+^CD8^+^ cells (10^9^/L)	0.53 (0.2-0.9)	1.21 (0.3-1.6)
γδ T cells (10^9^/L)	0.03	1.39
Naive CD4^+^ T cells (CD27^+^CD45RA^+^)		50% (>50%)
Naive CD8^+^ T cells (CD27^+^CD45RA^+^)		61% (>50%)
PHA, 0 μg/mL (mean CPM)	68	57
PHA, 4 μg/ml (mean CPM)	3,859 (>12,000)	10,909 (>12,000)
CD3 background (mean CPM)	167	183
CD3 stimulated (mean CPM)	484 (>7,500)	3,952 (>7,500)
*Candida* species background (mean CPM)	630	904
*Candida* species stimulated (mean CPM)	5,274 (>12,500)	9,252 (>12,500)
IgG (g/L)	7.2 (6-13)	1.8 (4.9-16.1)
IgG_1_ (g/L)	4.66 (3.1-8.9)	
IgG_2_ (g/L)	0.58 (1.4-5.5)	
IgG_3_ (g/L)	0.26 (0.04-1.07)	
IgG_4_ (g/L)	0.00 (0.01- 0.93)	
IgA (g/L)	2.3 (0.8-3.7)	1.0 (0.4-2.0)
IgM (g/L)	2.4 (0.4-2.2)	0.5 (0.5-2.0)
CD4^+^ TRECs per million T cells		19,057 (>20,000)
CD8^+^ TRECs per million T cells		34,125 (>20,000)
Naive B cells (IgD^+^IgM^+^CD27^−^)		98% (70% to 90%)
Anti-tetanus antibody (IU/mL)	<10 IU (undetectable)	0.28 (protective range)
Anti-pneumococcal antibody	<10 IU (undetectable); no response on immunization	220 before immunization/352 after immunization (640 units of range for unimmunized subject)
Anti-rubella antibody		4 (>10)
Anti–varicella zoster antibody		43 (>20)
Anti-mumps antibody		IgG positive
Anti-measles antibody		1.4 (detectable)
Anti-HiB antibody (μg/mL)		2.17 before immunization/>14 after immunization (optimal >1)

*CPM*, Counts per minute; *TRECs*, T-cell receptor excision circles.

**Table E2:** X-inactivation studies of the 3 generations in the family

Relationship	AR (CAG)n −*Hpa*II	AR (CAG)n +*Hpa*II	Conclusion
Son (grandson)	272	NA	High-risk X-chromosome
Mother of grandson	Whole blood 272, 292 T cells 272, 292	272, 292 272	Nonrandom X- inactivation in T cells
Grandfather	272		High-risk X-chromosome

**Fig 1:**
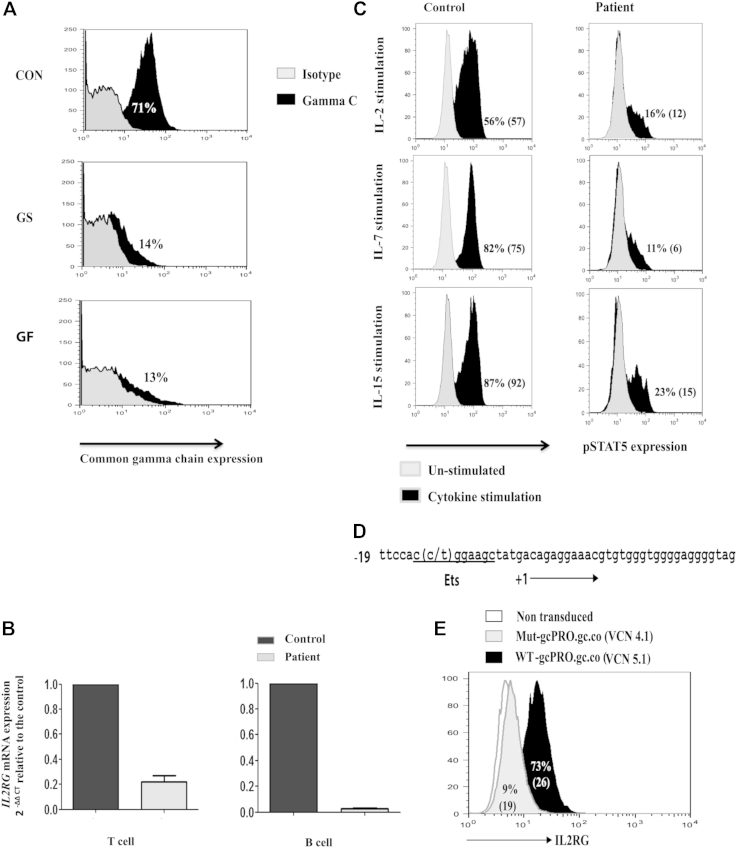
Reduced *IL2RG* expression and function. **A** and **B,** Common γc expression on lymphocytes (Fig 1, *A*) in a control subject *(CON)*, the grandson *(GS)*, and the grandfather *(GF)*, as well as *IL2RG* mRNA expression (Fig 1, *B*). **C,** Phosphorylated STAT5 *(pSTAT5)* expression after stimulation with cytokines *(dark gray)* or unstimulated *(light gray)*. Mean fluorescence intensities are shown in parentheses. **D,** Illustration of the point mutation in the *IL2RG* promoter with the ETS consensus sequence underscored. **E,** Expression of common γc after transduction of ED7R cells. The vector copy number *(VCN)* per cell is shown.

**Fig 2:**
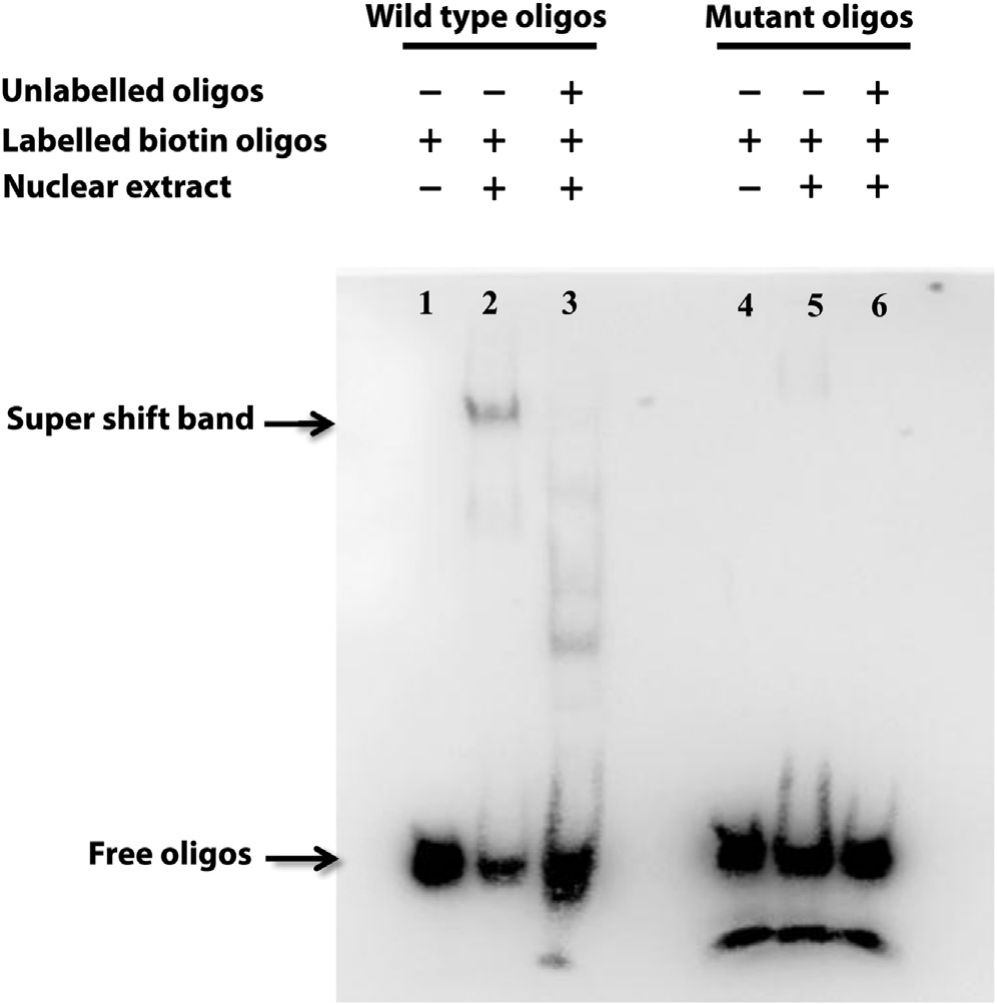
Electrophoretic mobility shift assay. Biotin-labeled wild-type or mutant oligonucleotides incubated without nuclear extracts (*lanes 1* and *4*), with nuclear extracts (*lanes 2*, *3*, *5*, and *6*), and in the absence (*lanes 2* and *5*) or presence (*lanes 3* and *6*) of an excess of unlabeled oligonucleotides. A supershift DNA/protein complex band is detected and marked. The free-labeled oligonucleotide is indicated.

## Methods

### Determination of *IL2RG* mRNA expression in peripheral blood by using quantitative RT-PCR

#### Cell separation from peripheral blood cells

A subset of T and B cells from control and patient samples were obtained by incubating PBMCs with anti-CD3 and anti-CD19 antibodies conjugated with magnetic microbeads (Miltenyi Biotec, Surrey, United Kingdom). Then the cells were separated with the autoMACS Pro Separator (Miltenyi Biotec).

#### Total RNA isolation and reverse transcription

Total RNA was extracted from sorted T and B cells by using the RNeasy kit (Qiagen, Hilden, Germany), according to the manufacturer's instructions. The cDNA was synthesized with the QuantiTect Reverse Transcription Kit (Qiagen). First, RNA (100 ng) was incubated with genomic DNA Wipeout Buffer at 42°C for 5 minutes. Then Reverse Transcription master mix containing RT primer and Quantiscript Reverse Transcriptase (Qiagen) was added to the RNA. The reaction was then incubated for 15 minutes at 42°C, followed by 3 minutes at 95°C to inactive reverse transcriptase.

#### Real-time quantitative PCR

Quantitative PCR was performed with an ABI 7000 Sequence Detection System (Applied Biosystems, Warrington, United Kingdom). The primer sequences for *IL2RG* were as follows: forward, 5-TGCTAAAACTGCAGAATCTGGT -3; reverse, 5-AGCTGGGATTCACTCAGTTTG-3. The *IL2RG* probe sequence was 5-CCTGGGCTCCAGAGAACCTAACA-TAMRA-3. The primer sequences for the human β-actin gene were as follows: forward, 5-TCACCCACACTGTGCCCATCTACGA-3; reverse, 5-CAGCGGAACCGCTCATTGCCAATGG-3. The β-actin probe sequence was 5-FAM-ATGCCCTCCCCCATGCCA TCCTGCGT-TAMRA-3. The average cycle threshold (Ct) values for *IL2RG* from quantitative PCR were normalized with the average Ct value of β-actin to give rise to the ΔCt, which is used to calculate the ΔΔCt value. Then the 2^−ΔΔCt^ values for healthy control subjects and patients were deduced from the ΔΔCt value. Quantitative PCR was run in duplicates in 3 independent experiments. Two different controls were used.

### Generation of the mutant *IL2RG* construct

The mutant *IL2RG* promoter was generated by using the QuikChange Lightning Site-Directed Mutagenesis Kit (Agilent Technologies, Wokingham, Berkshire, United Kingdom). The lentiviral vector containing the wild-type *IL2RG* promoter driving codon optimized *IL2RG* (lenti-gcPRO.gc.co)^E1^ was used as the parental sequence. The primer sequences used in PCR were as follows: forward, 5-ggttctttccactggaagctatgacag-3; reverse, 5-ctgtcatagcttccagtggaaagaacc-3. The PCR reaction was performed according to the manufacturer's instructions. PCR product was then incubated with *Dpn*I enzyme at 37°C for 5 minutes to digest parental plasmid DNA. Digested PCR product of 2 μL was transformed into XL10-Gold cells provided within the kit, according to the protocol. The plasmids prepared from single colonies were then sequenced to confirm introduction of the point mutation within the *IL2RG* promoter and to obtain the lenti-mut.gcPRO.gc.co construct.

### Lentivirus preparation

Lentivirus was produced by means of transient cotransfection of HEK293T cells with 3 plasmids, the lentiviral vectors for lenti-gcPRO.gc.co and lenti-mut.gcPRO.gc.co, pMD.G2 (envelope plasmid), and pCMV_8.91 (packaging plasmid, both produced by Plasmid Factory, Bielefeld, Germany), by using polyethylenimine (Sigma-Aldrich, St Louis, Mo), as previously described.^E2^

#### Determination of viral titer

Mouse fibroblast SC-1 cells were transduced with serial dilutions of virus. Genomic DNA was isolated from transduced cells by using the DNeasy Kit (Qiagen). Viral vector copy number was determined by using quantitative PCR. The primers for the lentiviral vector were as follows: forward, 5-CAGGACTCGGCTTGCTGAAG-3; reverse, 5-TCCCCCGCT TAATACTGACG-3. The probe for lentiviral vector was 5-FAM-CGCACGGCA AGA GGC GAG G TAMRA-3. The primer sequences for the mouse housekeeping titin gene *(Ttn)* were as follows: forward, 5-AAAACGAGCAGTGACCTGAGG-3; reverse, 5-TTCAG TCATGCTGCTAGCGC-3. The *Ttn* probe sequence was 5-FAM-TGCACGGAATCTCGTCTC AGTC-TAMRA-3.

### *IL2RG* expression analyzed by using fluorescence-activated cell sorting

#### *IL2RG* expression *in vitro* after lentivirus transduction

ED7R cells derived from an adult human T-cell leukemia line deficient in *IL2RG* gene expression were cultured in RPMI 1640 plus 10% FCS. The cells were seeded into 12-well plates (10^5^ cells/well) and transduced with the lenti-gcPRO.gc.co or lenti-MutgcPRO.gc.co viruses at different multiplicities of infection. On day 3 after transduction, half of the transduced cells were resuspended in 100 μL of fluorescence-activated cell sorting (FACS) buffer (0.5% BSA/PBS) containing 0.5 μL of anti–human IL2RG (BD Biosciences, San Jose, Calif) and incubated for 30 minutes at 4°C. The cells were then washed with FACS buffer, and *IL2RG* expression was analyzed by using FACS. Half of the transduced cells were pelleted for extraction of genomic DNA, and the vector copy numbers were determined by using quantitative PCR, as described above. The housekeeping gene albumin was used as the internal reference gene. The primers/probes used for the lentivirus vector is the same as the above. The primers for the human albumin gene were as follows: forward, 5-GCT GCT ATC TCT TGT GGG CTG T; reverse, 5-ACT CAT GGG AGC TGC TGG TTC. The albumin probe was 5′-VIC-CCT GTC ATG CCC ACA CAA ATC TCT CC-TAMRA-3.

#### *IL2RG* expression in patients

Three microliters of anti-human *IL2RG* (BD Biosciences) or matched isotype control (BD Biosciences) was added to 100 μL of blood incubated for 10 minutes at room temperature. Red blood cells were lysed with FACsLyse (BD Biosciences) for 10 minutes and then washed with Cell Wash (BD Biosciences) before being fixed (FACsFix, BD Bioscience). Ten thousand lymphocytes were acquired (FACSCalibur, BD Biosciences) and analyzed (CellQuest Pro, BD Biosciences).

#### FACS analysis of STAT5 tyrosine phosphorylation in T cells

IL-2 (1 × 10^4^ IU/mL; Chiron, Austin, Tex), IL-7 (100 ng/mL; R&D Systems, Minneapolis, Minn), or IL-15 (50 ng/mL, R&D Systems) was added to 100 μL of whole blood and placed at 37°C for 10 minutes to stimulate cells. Two milliliters of prewarmed FACsLyse/Fix (BD Biosciences) was then added to the blood, mixed, and placed at 37°C for 10 minutes. The cells were pelleted and washed once with STAT wash (PBS containing 1% FCS). The cells were resuspended in cold Perm Buffer III (BD Biosciences) and placed at 4°C for 30 minutes. The cells were then washed once with STAT wash before 5 μL of antibody (STAT5 ptyr and CD4 peridinin-chlorophyll-protein complex [PerCP]; BD Biosciences) was added, and the cells were incubated at room temperature for 30 minutes in the dark, washed with STAT wash, and fixed (FACsFix, BD Biosciences). Ten thousand lymphocyte events were acquired (FACsCalibur, BD Biosciences) and analyzed with CellQuest software (BD Biosciences).

### Electrophoretic mobility shift assay

Nuclear proteins were extracted from Jurkat and ED7R cells by using the NE-PER Nuclear and Cytoplasmic Extraction kit (Thermo Scientific, Waltham, Mass), according to the manufacturer's instructions. The extracted nuclear proteins were then placed in aliquots and stored at −80°C. A 38-bp oligonucleotide was chosen from the *IL2RG* promoter covering the Ets-binding region with the following sequences: wild-type, 5′ ATA AGG TTC TTT CCA CCG GAA GCT ATG ACA GAG GAA AC; mutant, 5′ ATA AGG TTC TTT CCA CTG GAA GCT ATG ACA GAG GAA AC. The oligonucleotides were synthesized and HPLC purified by Invitrogen (Carlsbad, Calif). Biotin labeling of the oligonucleotides was performed by using the Biotin 3′ End DNA Labeling Kit (Thermo Scientific), according to the manufacturer's instructions. The labeled oligonucleotides were purified with chloroform:isoamyl alcohol. An equal molar-labeled sense and antisense oligonucleotide was annealed in 10 mmol/L Tris, 1 mmol/L EDTA, and 50 mmol/L NaCl at 95°C for 5 minutes and then −1°C/cycle down to 59°C, followed for 30 minutes at 59°C, and then −1°C/cycle down to 20°C in the thermocycler. The same annealing process was also applied to the unlabeled oligonucleotides.

The binding assay was performed with the LightShift Chemiluminescent Electrophoretic Mobility Shift Assay Kit (Thermo Scientific), according to the manufacturer's instructions. The binding reaction contained 1 × binding buffer, 2.5% glycerol, 5 mmol/L MgCl_2_, 50 ng/μL Poly(dI*Dc) 0.05% NP-40, 4 pmol of unlabeled oligos (200 × excess), and 20 fmol of biotin-labeled oligonucleotides in a total volume of 20 μL. The reaction was incubated at room temperature for 20 minutes and then loaded onto a 6% DNA retardation TBE gel (Invitrogen). After electrophoresis, the gel was transferred to the nylon membrane (Invitrogen). The membrane was then UV cross-linked for 12 minutes and incubated in blocking buffer for 15 minutes, followed by incubation with streptavidin–horseradish peroxidase in blocking buffer. The membrane was then washed with 1 × wash buffer, followed by Substrate Equilibration Buffer (Thermo Scientific). Finally, the membrane was incubated with Substrate Working buffer for 5 minutes before exposure with the CCD camera.

### X-inactivation studies

X-inactivation studies were performed by the North East Regional Genetics Laboratory on DNA from whole blood and magnetically separated T cells (anti-CD3 beads and autoMACs, Miltenyi Biotec), as previously described.^E3^ This analyzes methylation at the androgen receptor locus. Briefly, extracted DNA was incubated with or without the restriction enzyme *Hpa*II, amplified, and analyzed on a sequencing gel. In female subjects with random X-inactivation, 2 bands are detected, and only 1 band is detected in those with nonrandom X-inactivation.

#### Patients' immunologic data

Immunophenotyping of lymphocyte populations was performed by using flow cytometry. Whole blood was labeled with combinations of mAbs conjugated with fluorescein isothiocyanate (FITC), phycoerythrin (PE), allophycocyanin (APC), PerCP, or fluorochrome combinations with cyanines (PerCP-Cy5.5, APC-Cy7, and PE-Cy7; BD Biosciences). Lymphocyte subsets were detected by using a 6-color multitest reagent containing CD3 FITC, CD16+CD56 PE, CD45 PerCP-Cy5.5, CD19 APC, CD4 PE-Cy7, and CD8-APC-Cy7. Naive, effector, and memory T-cell populations were detected with CD45RA FITC, CD27 PE, CD45 PerCP, and CD4 or CD8 APC. Naive, memory, and switched B-cell populations were detected by using CD19 PE-Cy7, CD27 PE, IgD FITC, and IgM Cy5. Staining was analyzed on a FACSCanto II flow cytometer (BD Biosciences).

Immunoglobulin levels were measured with a Dade Behring (Milton Keynes, United Kingdom) nephelometer (BNII), according to the manufacturer's instructions.

#### Proliferation assays

PBMCs (5 × 10^5^) were seeded onto a 96-well plate in RPMI containing 5% human AB serum. Mitogens (CD3 and PHA) or antigen (*Candida* species) were added to the wells in a final volume of 200 μL. A minimum of 3 replicates were performed on each sample, with a healthy control subject. Four days later, plates treated with mitogens were pulsed with 1 μCi/mL tritiated thymidine for 4 hours. Antigen plates were pulsed after 6 days, cells were harvested, and thymidine incorporation was measured on a scintillation counter.

### Whole-exome sequencing

Library preparation, exome capture, and sequencing have been done according to the manufacturers' instructions. For exome target enrichment, the Agilent SureSelect 50 Mb kit (Agilent, Santa Clara, Calif) was used. Sequencing was done with the Illumina HiSeq with 94-bp paired-end reads. Reads from raw FASTQ files were aligned to the hg19 reference genome by using NovoAlign, version 2.08.03 (Novocraft Technologies, Selangor, Malaysia). Duplicate reads were marked with picard tools mark duplicates. Calling was performed with the haplotype caller module of GATK (https://www.broadinstitute.org/gatk), creating gVCF formatted files for each sample. The individual gVCF files were combined into gVCF files containing 100 samples each. The final variant calling was performed with the GATK “GenotypegVCFs” module used jointly for all patients and control subjects. Variant quality scores were then recalibrated according to GATK best practices separately for indels and single nucleotide polymorphisms. Resulting variants were annotated with ANNOVAR.
